# The association of chorioamnionitis with intraventricular hemorrhage and long-term outcome in very preterm neonates

**DOI:** 10.3389/fped.2026.1829713

**Published:** 2026-06-15

**Authors:** Eftychia Drogouti, Dimitrios Rallis, Maria Lithoxopoulou, Georgios Kerpiniotis, Evangelia Aggeli, Ilias Chatziioannidis, Apostolos Athanasiadis, Christos Tsakalidis

**Affiliations:** 1 Second Department of Neonatology and NICU, Aristotle University of Thessaloniki, Thessaloniki, Greece; 2 First Department of Neonatology and NICU, Aristotle University of Thessaloniki, Thessaloniki, Greece; 3 Third Department of Obstetrics and Gynecology, Aristotle University of Thessaloniki, Thessaloniki, Greece

**Keywords:** cerebral palsy, funisitis, intrauterine infection, neurodevelopment, prematurity

## Abstract

**Objective:**

To evaluate the impact of chorioamnionitis on the incidence of intraventricular hemorrhage in preterm neonates and to assess its impact on neurodevelopmental outcomes at 24 months of age.

**Methods:**

We retrospectively reviewed the medical records of all neonates <32 weeks’ gestational age born to mothers with and without histological chorioamnionitis. At 24 months of age, we evaluated long-term neurodevelopmental outcomes with the Gross Motor Function Classification System (GMFCS) and the Modified Checklist for Autism in Toddlers (M-CHAT).

**Results:**

Among 91 neonates with a mean gestational age of 29.1(±2.2) weeks, 54% (49 newborns) were exposed to chorioamnionitis during pregnancy. Neonates born to mothers with chorioamnionitis were of significantly lower gestational age (28.0 ± 2.3 vs. 30.2 ± 1.3 weeks, *p* < 0.001) and birth weight (1,172 ± 400 g vs. 1,376 ± 409 g, *p* = 0.018) compared to neonates born to mothers without chorioamnionitis, respectively. Neonates of mothers with chorioamnionitis had an increased risk of intraventricular hemorrhage (OR 6.34, 95% CI 1.91–21.01, *p* = 0.003), retinopathy of prematurity (OR 2.48, 95% CI 1.13–5.46, *p* = 0.041), and bronchopulmonary dysplasia (OR 6.12, 95% CI 2.08–18.04, *p* = 0.001). After adjusting for gestational age and intraventricular hemorrhage, chorioamnionitis had an increased risk of abnormal M-CHAT (OR 4.52, 95% CI 1.12–18.20, *p* = 0.034) at 24 months of age.

**Conclusion:**

Very preterm neonates born to mothers with chorioamnionitis are at increased risk of severe intraventricular hemorrhage, retinopathy of prematurity, bronchopulmonary dysplasia, and an increased risk of abnormal M-CHAT at 24 months of age.

## Introduction

Chorioamnionitis, defined as inflammation of the chorion or amniotic membrane, often due to ascending bacterial infection, is a leading contributor to preterm birth (1). In a previous report from our site, we reported that amongst infants with suspected chorioamnionitis, the diagnosis was partially supported by histological confirmation in pregnancies of a lower gestational age (2). Preterm infants are at high risk for brain injury, among which intraventricular hemorrhage is one of the most serious. Intraventricular hemorrhage frequently originates in the germinal matrix, a fragile, highly vascularized region in the preterm brain (3). Severe intraventricular hemorrhage is strongly associated with mortality and long-term neurodevelopmental impairments (4). Given that chorioamnionitis often triggers systemic and fetal inflammatory responses, researchers have hypothesized that chorioamnionitis may predispose to intraventricular hemorrhage through multiple pathophysiological pathways, with downstream implications for neurodevelopment (5). Biological plausibility is strong, given inflammatory disruption of vascular integrity, hemodynamic instability, and coagulation abnormalities. Because intraventricular hemorrhage is itself strongly linked to neurodevelopmental impairment (e.g., cerebral palsy, cognitive delay), chorioamnionitis may exert its long-term effects largely via hemorrhagic injury. Moreover, studies adjusting for confounders show that chorioamnionitis may also have a subtler independent impact on cognition, potentially mediated by chronic neuroinflammation (6). However, not all studies are consistent: some fail to show elevated intraventricular hemorrhage or neurodevelopmental deficits in chorioamnionitis-exposed infants after adjustment (7), perhaps due to heterogeneity in definitions (clinical vs. histologic chorioamnionitis), timing of intraventricular hemorrhage assessment, or differences in postnatal care. Moreover, funisitis (histological marker of fetal inflammatory response) has not always been associated with higher intraventricular hemorrhage risk compared to chorioamnionitis without funisitis, suggesting that fetal inflammation *per se* may not fully account for hemorrhagic risk (1, 8).

While the association between chorioamnionitis and intraventricular hemorrhage has been previously described in several cohort studies and meta-analyses, substantial heterogeneity persists regarding the magnitude and independence of this association after adjustment for gestational age and other perinatal confounders. Furthermore, comparatively fewer studies have evaluated both neonatal morbidities and longer-term neurodevelopmental outcomes within the same histologically confirmed cohort. Therefore, this study aimed to evaluate the association between maternal histological chorioamnionitis and the development of intraventricular hemorrhage and other neonatal morbidities, namely bronchopulmonary dysplasia and retinopathy of prematurity, in very preterm neonates, as well as its association with neurodevelopmental outcomes at 24 months of age.

## Methods

A retrospective cohort study was conducted in a university perinatal center over a period of 3 years (2020–2022). The medical records of neonates who were born <32 weeks of gestational age were reviewed, and neonates were classified into those born to mothers with histological chorioamnionitis and those born to mothers without evidence of chorioamnionitis. Maternal chorioamnionitis was defined according to the ACOG criteria (9); Suspected intraamniotic infection was based on clinical criteria, which included maternal intrapartum fever and one or more of the following: maternal leukocytosis, purulent cervical drainage, or fetal tachycardia. Confirmed intraamniotic infection was based on a positive amniotic fluid test result (gram stain, glucose level, or culture results consistent with infection) or placental pathology demonstrating histologic evidence of placental infection or inflammation (9). In our study, maternal chorioamnionitis was defined exclusively according to histopathological placental examination. Placental pathology reports demonstrating histologic evidence of placental inflammation or infection were considered diagnostic of histological chorioamnionitis. Histopathological evaluation was performed according to routine institutional pathology reporting practices. Maternal inflammatory response and, when available, fetal inflammatory response/funisitis findings were recorded from placental pathology reports. Because of the retrospective nature of the study, detailed staging and grading according to the Amsterdam Placental Workshop Group (10) or Redline classification (11) systems were not consistently available for all placentas. Infants without placental histologic examination or incomplete medical records were excluded. The data were anonymously recorded, and the study was approved by the ethical committee of the institution (No 2026-Β230-530/11.02.26).

Maternal data were retrospectively collected, including maternal age, mode of delivery, premature rupture of membranes, parity, antenatal administration of corticosteroids, maternal antibiotic administration, and maternal inflammatory markers, including C-reactive protein and white blood cell count prior to delivery. Neonatal data included gestational age, birthweight, sex, intrauterine growth restriction, group B Streptococcus colonization, complete course of antenatal steroids, delivery mode, need for resuscitation in the delivery room, and Apgar score. We also recorded neonatal morbidities including respiratory distress syndrome, pneumothorax, pulmonary hemorrhage, the need for inotropes administration, patent ductus arteriosus, early and late-onset sepsis, necrotizing enterocolitis, retinopathy of prematurity, intraventricular hemorrhage, and periventricular leukomalacia, based on Vermont – Oxford Network criteria (12), seizures, the duration of invasive and non-invasive mechanical ventilation, rates of bronchopulmonary dysplasia and grade based on standard criteria (13), postmenstrual age and birthweight on discharge, and survival. The long-term neurodevelopmental outcome was evaluated at 24 months of age with the Gross Motor Function Classification System (GMFCS) and the Modified Checklist for Autism in Toddlers (M-CHAT). The GMFCS is considered a standardized way to describe gross motor abilities and a reliable measure for functional severity (14), while the M-CHAT is the most commonly used toddler screener for autism spectrum disorder; in a recent review including 50 studies, the pooled sensitivity of M-CHAT was 0.83 (95% CI, 0.77–0.88), and the pooled specificity was 0.94 (95% CI, 0.89–0.97) (15).

The primary outcome included the association of chorioamnionitis with the development of intraventricular hemorrhage and common neonatal morbidities, including bronchopulmonary dysplasia and retinopathy of prematurity. The secondary outcomes included the correlation of an abnormal M-CHAT with maternal chorioamnionitis, adjusted for gestational age and intraventricular hemorrhage.

### Statistical analysis

Descriptive statistics were calculated for maternal and neonatal characteristics. The normality of the distributions of continuous variables was assessed with the Shapiro–Wilk normality test. Continuous variables were expressed as mean ± standard deviation or median (interquartile range), and categorical variables as *n* (percentage %). Comparisons between continuous variables were performed with the student's t-test, or the non-parametric Mann–Whitney test, as appropriate, whereas comparisons between categorical variables utilized the chi-square test or Fisher's exact test.

A multivariate logistic regression analysis was performed to examine the association of intraventricular hemorrhage, bronchopulmonary dysplasia, retinopathy of prematurity, and seizures with maternal chorioamnionitis, adjusted for gestational age. Given the relatively limited sample size and event rates, additional adjustment for multiple postnatal variables was avoided to minimize model overfitting. A separate multivariate logistic regression analysis examined the association of abnormal M-CHAT findings at 24 months of age with maternal chorioamnionitis after adjustment for gestational age and intraventricular hemorrhage. The Bonferroni correction for multiple testing was utilized. Collinearity was examined with a correlation matrix, and variables with significant collinearity, such as gestational age and birth weight, were examined separately in the model. All tests were two-sided, and a *p*-value less than 0.05 was considered statistically significant (alpha 0.05). The data were analyzed using SPSS Statistics Version 25.0 (IBM SPSS Statistics for Windows, Version 24.0. Armonk, NY, US).

## Results

**Figure 1:**
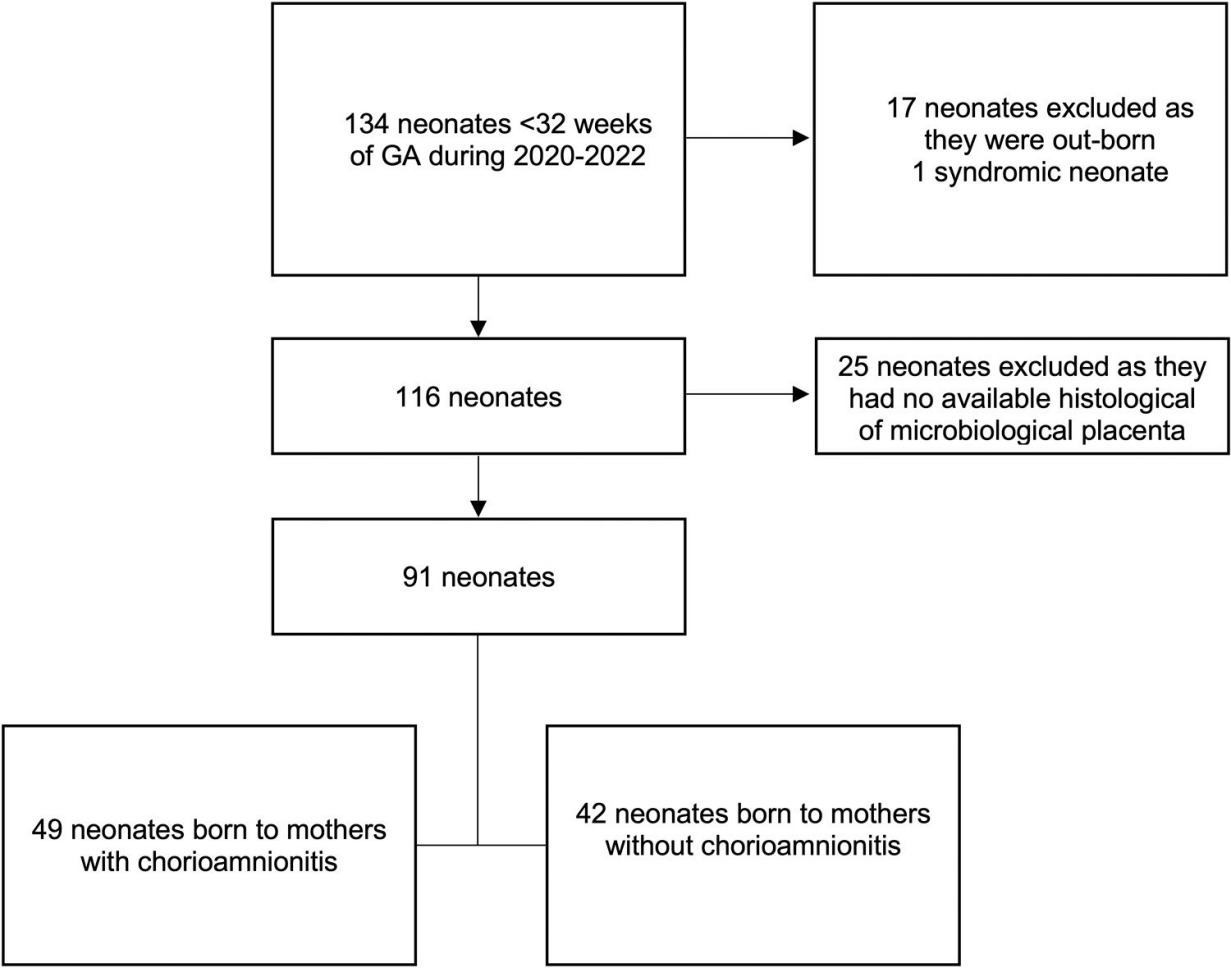
Flow chart of the study population.

In a sample of 91 neonates with a mean gestational age of 29.1 ± 2.2 weeks and birth weight of 1,266 ± 415 g, 54% (49 newborns) were exposed to chorioamnionitis during pregnancy (Figure 1). Neonates born to mothers with chorioamnionitis were of significantly lower gestational age (28.0 ± 2.3 vs. 30.2 ± 1.3 weeks, *p* < 0.001) and birth weight (1,172 ± 400 g vs. 1,376 ± 409 g, *p* = 0.018) compared to neonates born to mothers without chorioamnionitis, respectively (Table 1). Maternal antenatal antibiotic administration was significantly more frequent in the chorioamnionitis group compared to the non-chorioamnionitis group (56% vs. 28%, *p* = 0.005). Maternal C-reactive protein levels prior to delivery were higher in mothers with histological chorioamnionitis compared to mothers without chorioamnionitis [1.54 (0.51–3.30) vs. 0.91 (0.53–1.20) mg/dL, *p* = 0.001]. Moreover, neonates born to mothers with chorioamnionitis, compared to those without chorioamnionitis, developed at higher rates intraventricular hemorrhage (32% compared to 10%, *p* = 0.011), retinopathy of prematurity (16% compared to 2%, *p* = 0.033), and bronchopulmonary dysplasia (36% compared to 14%, *p* = 0.013). Of the 91 neonates, 4 (4%) neonates born to mothers with chorioamnionitis had a severe intraventricular hemorrhage (III-IV) (Table 2).

**Table 1:** Perinatal characteristics of the study cohort and between neonates born to mothers with chorioamnionitis vs. neonates born to mothers without chorioamnionitis.

Variables	Total cohort (*n*-91)	Chorioamnionitis (*n* = 49)	Non-chorioamnionitis (*n* = 42)	*p*
Gestational age, weeks	29.1 ± 2.2	28.0 ± 2.3	30.2 ± 1.3	<0.001
Birth weight, g	1,266 ± 415	1,172 ± 400	1,376 ± 409	0.018
Sex, male	49 (54%)	22 (44%)	27 (64%)	0.141
Maternal age, years	32.6 ± 8.1	33.8 ± 8.0	31.0 ± 8.0	0.194
*In-vitro* fertilization	22 (24%)	9 (18%)	13 (31%)	0.226
Primiparity	62 (68%)	38 (76%)	24 (57%)	0.082
Cesarean section	84 (92%)	43 (86%)	41 (98%)	0.118
Prolonger rupture of membranes	21 (23%)	14 (28%)	7 (17%)	0.217
Antenatal antibiotic administration	40 (44%)	28 (56%)	12 (28%)	0.005
Antenatal steroid administration	68 (74%)	35 (70%)	33 (79%)	0.989
Maternal C-reactive protein, mg/dL	1.04 (0.52–1.87)	1.54 (0.51–3.30)	0.91 (0.53–1.20)	0.001
Maternal white blood cells, cells/*μ*L	13,100 (10,500–16,200)	15,300 (11,300–17,900)	12,400 (10,100–13,100)	0.037
Apgar 1st minute	7 (6–8)	7 (6–8)	7 (7–8)	0.194
Apgar 5th minute	8 (8–9)	8 (8–9)	8 (8–9)	0.997

Continuous variables are expressed as mean ± standard deviation or median (interquartile range). *P*-value of the student's t-test or the Mann–Whitney U test. Categorical variables are expressed as *n* (%). *P*-value of the chi-square test.

**Table 2:** Outcomes of the study cohort and between neonates born to mothers with chorioamnionitis vs. neonates born to mothers without chorioamnionitis.

Variables	Total cohort (*n*-91)	Chorioamnionitis (*n* = 49)	Non-chorioamnionitis (*n* = 42)	p
Respiratory distress syndrome	57 (63%)	31 (62%)	26 (62%)	0.819
Transient tachypnea	5 (5%)	2 (4%)	3 (7%)	0.664
Persistent pulmonary hypertension	2 (2%)	1 (2%)	1 (2%)	0.989
Emphysema	3 (3%)	3 (6%)	-	0.242
Early-onset sepsis	12 (13%)	9 (18%)	3 (7%)	0.212
Late-onset sepsis	36 (40%)	19 (38%)	17 (40%)	0.998
Patent ductus arteriosus	20 (22%)	12 (24%)	8 (19%)	0.458
Necrotizing enterocolitis	5 (5%)	2 (4%)	3 (7%)	0.660
Retinopathy of prematurity	9 (10%)	8 (16%)	1 (2%)	0.033
Bronchopulmonary dysplasia	24 (26%)	18 (36%)	6 (14%)	0.013
Intraventricular hemorrhage	20 (22%)	16 (32%)	4 (10%)	0.011
Severe intraventricular hemorrhage	4 (4%)	4 (8%)	-	0.121
Intraventricular hemorrhage, grade				0.011
I	13 (13%)	9 (18%)	4 (10%)	
II	3 (3%)	3 (6%)	-	
III	2 (2%)	2 (4%)	-	
IV	2 (2%)	2 (4%)	-	
Periventricular leukomalacia	1 (1%)	1 (2%)	-	0.987
Seizures	4 (4%)	4 (8%)	-	0.121
Survival	83 (91%)	42 (84%)	41 (98%)	0.065
Hypotonia at discharge	31 (34%)	19 (38%)	12 (29%)	0.171
MRI, abnormal	13 (14%)	8 (16%)	5 (12%)	0.540
GMFCS, abnormal	3 (3%)	3 (6%)	-	0.245
M-CHAT, abnormal	11 (12%)	9 (18%)	2 (5%)	0.047

Categorical variables are expressed as *n* (%). *P*-value of the chi-square test.

MCHAT, Modified Checklist for Autism in Toddlers; GMFCS, Gross Motor Function Classification System; MRI, Magnetic Resonance Image.

Neonates born to mothers with chorioamnionitis had an abnormal brain MRI at term-equivalent age in 16% vs. 12% of neonates born to mothers without (*p* = 0.540). At 24 months of age, three (6%) neonates of mothers with chorioamnionitis had an abnormal GMFCS (*vs* no neonate without chorioamnionitis, *p* = 0.245), and nine (18%) neonates with *vs* two (5%) without chorioamnionitis had abnormal M-CHAT (*p* = 0.047) (Table 2).

After adjusting for gestational age, neonates born to mothers with chorioamnionitis had an increased risk of intraventricular hemorrhage (OR 6.34, 95% CI 1.91–21.01, *p* = 0.003), retinopathy of prematurity (OR 2.48, 95% CI 1.13–5.46, *p* = 0.041), and bronchopulmonary dysplasia (OR 6.12, 95% CI 2.08–18.04, *p* = 0.001), compared to neonates born to mothers without chorioamnionitis. Also, after adjusting for gestational age and intraventricular hemorrhage, chorioamnionitis had an increased risk of abnormal M-CHAT (OR 4.52, 95% CI 1.12–18.20, *p* = 0.034) at 24 months of age (Table 3).

**Table 3:** Multivariate logistic regression analysis of the association of intraventricular hemorrhage, bronchopulmonary dysplasia, retinopathy of prematurity, and seizures with chorioamnionitis, adjusted for gestational age, and abnormal M-CHAT at 24 months of age adjusted for gestational age and intraventricular hemorrhage.

Variables	OR	95% CI	*p*
Intraventricular hemorrhage			
Chorioamnionitis	6.34	1.91–21.01	0.003
Gestational age	0.85	0.79–0.92	0.001
Retinopathy of prematurity			
Chorioamnionitis	2.48	1.13–5.46	0.041
Gestational age	0.76	0.61–0.94	0.014
Bronchopulmonary dysplasia			
Chorioamnionitis	6.12	2.08–18.04	0.001
Gestational age	0.87	0.82–0.93	<0.001
Abnormal M-CHAT			
Chorioamnionitis	4.52	1.12–18.20	0.034
Gestational age	0.83	0.75–0.91	0.005
Intraventricular hemorrhage	2.03	0.41–10.09	0.384

MCHAT, Modified Checklist for Autism in Toddlers; GMFCS, Gross Motor Function Classification System; MRI, Magnetic Resonance Image; OR, odds ratio; CI, confidence intervals.

## Discussion

The findings of our study suggested that preterm neonates born to mothers with histological chorioamnionitis are at increased risk of intraventricular hemorrhage, retinopathy of prematurity, and bronchopulmonary dysplasia. Also, preterm neonates born to mothers with histological chorioamnionitis are at increased risk of an abnormal M-CHAT at 24 months of age, after adjustment for gestational age and intraventricular hemorrhage. While the association between chorioamnionitis and intraventricular hemorrhage has been previously described, our study contributes contemporary data from a histologically confirmed cohort and additionally explores early neurodevelopmental screening outcomes.

Our findings align with previous evidence suggesting a strong association between chorioamnionitis and intraventricular hemorrhage. A systematic review and meta-analysis by Villamor-Martinez et al. included 85 studies and over 46,000 infants; exposure to chorioamnionitis was associated with significantly increased odds of all grades of intraventricular hemorrhage (OR 1.88, 95% CI 1.61–2.19), of mild intraventricular hemorrhage (grades I–II; OR 1.69, 95% CI 1.22–2.34), and of severe intraventricular hemorrhage (grades III–IV; OR 1.62, 95% CI 1.42–1.85) (1). Importantly, the association persisted even after adjusting for confounders such as gestational age and birth weight, suggesting that chorioamnionitis imparts risk beyond simply inducing earlier delivery (1). A broader meta-analysis of antenatal infection and intraventricular hemorrhage supports similar findings: antenatal infection (most commonly chorioamnionitis) was associated with a higher risk of both mild (OR 1.95) and severe (OR 2.65) intraventricular hemorrhage, independent of gestational age (meta-analysis of 23 cohort studies with 13,605 infants; OR for infection overall 2.18) (16). Additionally, a cohort study of infants born to mothers with preterm premature rupture of membranes found that maternal chorioamnionitis was an independent risk factor for intraventricular hemorrhage, after controlling for gestational age and other perinatal variables (17). Of note, an earlier, smaller study examining extremely preterm infants (<28 weeks of gestational age) found no significant difference in early-onset intraventricular hemorrhage (within 72 h) between infants with histological chorioamnionitis and those without (14.5% vs. 18.2%; *p* = 0.48), disputing the association (18). Nevertheless, the majority of the evidence and the weight of evidence from large-scale meta-analyses support a positive and clinically meaningful association.

Several plausible mechanistic pathways connect chorioamnionitis to intraventricular hemorrhage. First, systemic fetal inflammation (i.e., the fetal inflammatory response syndrome) leads to elevated proinflammatory cytokines—such as interleukin-6 (IL-6), IL-1β, and tumor necrosis factor-α (TNF-α)—in the fetal circulation (19). These cytokines can impair vascular stability by weakening endothelial tight junctions, promoting endothelial activation, and perturbing vascular reactivity (19). The germinal matrix microvasculature in preterm infants is particularly fragile, lacking fully mature autoregulation, making it vulnerable to such inflammatory insults. Second, inflammation may disrupt cerebral autoregulation and hemodynamics. Inflammatory mediators may induce vascular tone dysregulation, hypotension, or fluctuations in cerebral blood flow (3, 20). Meta-analytic data revealed a correlation between chorioamnionitis, patent ductus arteriosus, and intraventricular hemorrhage; a hemodynamically significant patent ductus arteriosus might further destabilize cerebral circulation, increasing hemorrhage risk (1). Third, inflammation can affect coagulation; chorioamnionitis has been associated with coagulopathy in neonates, including thrombocytopenia and altered clotting factors, which may exacerbate hemorrhagic vulnerability (21). Finally, direct neuroinflammatory effects in the brain may contribute: cytokines can cross or signal at the blood-brain barrier, activate microglia, and trigger endothelial and glial cell adhesion molecule expression, leading to leukocyte–platelet interactions, endothelial damage, and increased blood viscosity or rheologic instability (22, 23). Additional studies support this association. Huang et al. demonstrated in a meta-analysis of 23 cohort studies including 13,605 infants that antenatal infection was independently associated with both mild and severe intraventricular hemorrhage (16). Similarly, Lu et al. identified maternal chorioamnionitis as an independent predictor of intraventricular hemorrhage in infants born after preterm premature rupture of membranes (17). Although some smaller studies failed to demonstrate a significant association after adjustment (24, 25), the overall body of evidence supports a biologically plausible relationship between antenatal inflammation and hemorrhagic brain injury in very preterm neonates (1, 8).

Apart from intraventricular hemorrhage, chorioamnionitis was also associated with bronchopulmonary dysplasia and retinopathy of prematurity in our cohort. Chorioamnionitis is repeatedly linked to adverse neonatal outcomes, including bronchopulmonary dysplasia and—less consistently—retinopathy of prematurity. Multiple systematic reviews and meta-analyses report that chorioamnionitis exposure increases the risk of bronchopulmonary dysplasia in very preterm infants, although much of this association is mediated by earlier gestational age and greater respiratory immaturity among chorioamnionitis-exposed neonates (26). The biological plausibility for chorioamnionitis-associated bronchopulmonary dysplasia includes antenatal inflammatory priming of the fetal lung, dysregulated pulmonary vascular and alveolar development, and interaction with postnatal insults (ventilation, oxygen). Recent cohort and histopathology studies support an independent effect of severe histologic chorioamnionitis on later bronchopulmonary dysplasia risk, but the effect size varies across studies after adjusting for confounders (27). For retinopathy of prematurity, systematic reviews show mixed findings: several meta-analyses found an association between chorioamnionitis and increased retinopathy of prematurity risk, but heterogeneity and confounding (prematurity, oxygen therapy, sepsis) complicate causal inference (28, 29). Some pooled analyses suggest a modest increased odds of any-stage retinopathy of prematurity with chorioamnionitis exposure, while others conclude the relationship weakens after rigorous adjustment (29). Interestingly, a recent meta-analysis revealed a negative association between chorioamnionitis and retinopathy of prematurity, suggesting a possible protective effect of chorioamnionitis against severe ROP, potentially mediated by inflammatory markers (30). In our study, regression analyses were adjusted primarily for gestational age because of sample size limitations and concern for model overfitting. Therefore, residual confounding from postnatal respiratory support, oxygen exposure, steroid administration, and sepsis cannot be excluded, and these findings should be interpreted cautiously.

Another significant finding in our study is that chorioamnionitis is associated with an abnormal M-CHAT at 24 months of age. The association of chorioamnionitis with the neurodevelopmental outcome was significant even after adjusting for the gestational age and the development of intraventricular hemorrhage. Intraventricular hemorrhage, especially high-grade (Grade III–IV), is a robust predictor of adverse neurodevelopmental outcomes. A recent study of preterm infants assessing early neurodevelopment (3–4 months corrected age) found that severe intraventricular hemorrhage increased the odds of cerebral palsy/high-risk of cerebral palsy diagnosis (adjusted OR 6.07 for severe intraventricular hemorrhage; OR 15.3 for severe brain injury, including intraventricular hemorrhage or cystic periventricular leukomalacia) (31). These early neurological assessments often portend later motor disability, cognitive deficits, and cerebral palsy. Beyond hemorrhagic injury, chorioamnionitis-related inflammation may independently impair neurodevelopment. One large multicentre cohort of 350 preterm infants assessed the association of histologic chorioamnionitis with neurodevelopment at 18–24 months corrected age (7). While chorioamnionitis was not significantly associated with intraventricular hemorrhage or punctate white matter injury after risk adjustment, there was a small, marginal reduction in cognitive performance (adjusted Bayley-III cognitive score difference −3.0, 95% CI −6.4 to 0.4), though motor scores did not differ significantly (adjusted difference −2.2, 95% CI −5.6 to 1.3) (7). Also, a previous study of 197 infants revealed that histological chorioamnionitis was associated with poorer cognitive outcome and weaker memory and learning function at 5 years of age (32). On the other hand, a study of 177 very low-birth-weight infants suggested a higher incidence of intraventricular hemorrhage and retinopathy of prematurity but similar neurodevelopmental scores and risk of cerebral palsy associated with chorioamnionitis (33). These findings suggest that although chorioamnionitis may increase intraventricular hemorrhage risk, its direct effect on early neurodevelopment, once perinatal confounders are considered, might be more modest than previously thought. Although we observed an association between chorioamnionitis and abnormal M-CHAT findings at 24 months of age, the relatively small number of abnormal outcomes and the wide confidence intervals suggest substantial statistical imprecision. Furthermore, M-CHAT and GMFCS represent screening and functional assessment tools rather than comprehensive neurodevelopmental testing. Consequently, these findings should be interpreted cautiously and considered exploratory and hypothesis-generating. Larger prospective studies with standardized neurodevelopmental assessments and longer follow-up are required to clarify the long-term neurodevelopmental impact of histological chorioamnionitis.

Overall, the evidence indicates that chorioamnionitis is a significant risk factor for intraventricular hemorrhage in preterm infants, with meta-analyses showing consistently elevated odds across intraventricular hemorrhage severity grades (1, 16). Recognizing chorioamnionitis as a risk factor for intraventricular hemorrhage has practical implications. Obstetric management of suspected or confirmed chorioamnionitis should remain aggressive, including timely antibiotic therapy and judicious decisions about the timing of delivery. In the neonatal period, infants born after chorioamnionitis exposure may benefit from heightened surveillance (e.g., early cerebral ultrasound) and rigorous strategies to maintain hemodynamic stability, minimize fluctuations in cerebral blood flow, and avoid coagulopathy. Furthermore, identifying chorioamnionitis-exposed infants, especially those with biomarkers of fetal inflammatory response, might help neonatologists stratify risk and apply neuroprotective strategies (such as early interventions, neuro-monitoring, or inflammation-modulating therapies).

The limitations of our study should be acknowledged. First, this was a single-centre retrospective study, and therefore, the findings should be interpreted cautiously before generalization to broader populations. Second, the number of severe intraventricular hemorrhage cases and abnormal neurodevelopmental outcomes was limited, reducing statistical power for these clinically important outcomes. Third, although analyses were adjusted for gestational age, residual confounding related to postnatal respiratory support, oxygen exposure, postnatal steroid administration, and sepsis may have influenced bronchopulmonary dysplasia and retinopathy of prematurity outcomes. Fourth, detailed placental histopathological staging and grading according to standardized international classification systems were not consistently available because of the retrospective design. Finally, neurodevelopmental assessment relied on screening and functional assessment tools rather than formal comprehensive neurodevelopmental testing. Only a few neonates reported an abnormal MRI and GMFCS score at 12 months of age, which could be attributed to the time of the follow-up assessment. The clinical findings of cerebral palsy are usually evident later than 24 months of age, and thus, some neonates might not have developed abnormal clinical signs at the time of the assessment.

In conclusion, histological chorioamnionitis appears to be associated with increased risk of intraventricular hemorrhage, bronchopulmonary dysplasia, and retinopathy of prematurity in very preterm neonates, independent of gestational age. Exposure to histological chorioamnionitis was additionally associated with abnormal M-CHAT findings at 24 months of age; however, these neurodevelopmental findings require cautious interpretation and validation in larger prospective studies with formal long-term neurodevelopmental assessment. Further research is needed to clarify the underlying inflammatory and hemodynamic mechanisms linking chorioamnionitis with neonatal brain injury and adverse developmental outcomes. Longitudinal neurodevelopmental follow-up into childhood is also essential to assess whether early subtle deficits in cognition or behavior persist or evolve. Neuroimaging studies might help elucidate structural changes (e.g., white matter injury) in relation to chorioamnionitis and intraventricular hemorrhage.

## Data Availability

The raw data supporting the conclusions of this article will be made available by the authors, without undue reservation available upon reasonable request.
